# Elevation of Fatty Acid Biosynthesis Metabolism Contributes to Zhongshengmycin Resistance in *Xanthomonas oryzae*

**DOI:** 10.3390/antibiotics10101166

**Published:** 2021-09-25

**Authors:** Qiaoxing Wang, Meiyun Lin, Peihua Shen, Yi Guan

**Affiliations:** Fujian Key Laboratory of Marine Enzyme Engineering, College of Biological Science and Engineering, Fuzhou University, Fuzhou 350116, China; N190820026@fzu.edu.cn (Q.W.); 200827031@fzu.edu.cn (M.L.); 200827045@fzu.edu.cn (P.S.)

**Keywords:** *Xanthomonas oryzae*, antibiotics, antibiotic-resistant, zhongshengmycin, fatty acid biosynthesis metabolism

## Abstract

*Xanthomonas oryzae* severely impacts the yield and quality of rice. Antibiotics are the most common control measure for this pathogen; however, the overuse of antibiotics in past decades has caused bacterial resistance to these antibiotics. The agricultural context is of particular importance as antibiotic-resistant bacteria are prevalent, but the resistance mechanism largely remains unexplored. Herein, using gas chromatography–mass spectrometry (GC–MS), we demonstrated that zhongshengmycin-resistant *X. oryzae* (Xoo-Rzs) and zhongshengmycin-sensitive *X. oryzae* (Xoo-S) have distinct metabolic profiles. We found that the resistance to zhongshengmycin (ZS) in *X. oryzae* is related to increased fatty acid biosynthesis. This was demonstrated by measuring the Acetyl-CoA carboxylase (ACC) activity, the expression levels of enzyme genes involved in the fatty acid biosynthesis and degradation pathways, and adding exogenous materials, i.e., triclosan and fatty acids. Our work provides a basis for the subsequent control of the production of antibiotic-resistant strains of *X. oryzae* and the development of coping strategies.

## 1. Introduction

Rice is one of the most widely cultivated and economically important crops in the world and is related to the survival of half of the world’s population [1]. However, the quality and yield of rice are threatened by *Xanthomonas oryzae*, a Gram-negative bacterium. *X. oryzae* is characterized as being short and rod-shaped, with a layer of mucinous extracellular polysaccharide on the surface, which makes its colony yellow and shiny [2,3]. *Xanthomonas oryzae* pv. *oryzae* and *Xanthomonas oryzae* pv. *oryzicola* are two main pathovars of *X. oryzae* that infect rice leaves through wounds or stomata, and cause rice leaf blight and rice leaf streak [4,5,6]. These two diseases often occur in rice-growing areas, such as Japan, India, and China, and are explosive and destructive in nature, sometimes leading to more than a 50% reduction in production or, in extreme cases, a harvestless year [3,7].

In the field of pathogen control, agricultural, biological, and chemical measures are the three main strategies [8,9]. As compared with biological and chemical measures, agricultural control is a more traditional and simpler way to avoid crop diseases, but these methods are often limited by the labor force, planting area, and the climatic environment. Genetic breeding is the most direct measure to control *X. oryzae.* This involves selecting natural mutations that confer resistance to the infection. Importantly, the selected strains can then be further propagated for large-scale production [10]. Nonetheless, this process is time-consuming and labor-intensive. To fill this gap, chemical measures, and antibiotics in particular, are widely adopted to treat *X. oryzae* infection. However, the misuse/overuse of antibiotics causes widespread antibiotic resistance, and these bacteria are insensitive to the currently available antibiotics. Recently, antibiotic-resistant *X. oryzae* strains were identified in a number of studies [11,12,13]. Thus, the mechanisms underlying drug resistance have aroused the interest of researchers.

Zhongshengmycin (ZS herein) is a low toxicity aminoglycoside antibiotic produced by *Streptomyces lavendulae* var. *Hainanensis* in Hainan, which is widely used as a chemical bactericide in the control of phytopathogens. Previous studies showed that ZS can be used to control vegetable bacterial diseases, rice bacterial leaf blight, and fruit tree diseases [14,15]. Additionally, ZS has good herbicidal and fungicidal properties and has been used as a control agent with which to study the activity of many new compounds [16,17]. Currently, ZS is applied in 10 provinces in Southeast Asia for the control of plant bacterial diseases, and economic benefits have been reported [18]. However, as a result of drug resistance, the control effect of ZS on rice bacterial blight has become negligible, and it will take a long time for new effective agents to be developed. Therefore, it is necessary to study the resistance mechanism of *X. oryzae* to ZS to restore ZS-mediated efficacy.

Four main mechanisms of bacterial antibiotics resistance have been revealed: the production of inactivating enzymes, changing the target of antimicrobial agents, activating the efflux pump system, and changing the permeability of the outer membrane [19,20,21]. Recently, the key role that metabolism plays in bacterial antibiotics resistance has been demonstrated [22,23,24]. For example, a low abundance of respiratory nitrate reductase is essential for *Escherichia coli* resistance to aminoglycoside and cephalosporin [25]. The low efficiency of the central carbon and energy metabolism was shown to mediate the resistance of *Vibrio alginolyticus* to levofloxacin and ceftazidime [26,27]. Drug-resistant bacteria can provide protection for other more vulnerable bacteria through the secondary metabolite indole so as to improve the survival ability of the whole population in stressful environments [28]. The existing research on *X. oryzae* is mainly focused on the effects that biological agents, nano agents, and gene knockout have on its virulence and control [8,29,30,31]; however, studies concerning the mechanism of drug resistance of *X. oryzae* from the perspective of metabolomics are lacking.

Metabolomics is a newly developed discipline and has become a research hotspot in recent years. Gas chromatography–mass spectrometry (GC–MS) is a useful technical method to collect the metabolite spectrum of samples through full spectrum analyses. Recently, metabolomics assays have been extensively used to analyze the metabolic mechanism of microbial resistance [32,33]. For instance, alanine metabolism promoted the production of reactive oxygen species, while glucose increased the metabolism of glycine, serine, and threonine, which promoted the killing of kanamycin-mediated antibiotic-resistant bacteria [34,35]. Fructose, glycine, and serine were the key metabolites promoting the growth and antifungal activity of *Penicillium citrinum* W1 [36,37]. Therefore, the GC–MS-based metabolomics method is an ideal approach with which to explore the metabolic mechanism of *X. oryzae* resistance to ZS.

In the present study, we compared the metabolomes of zhongshengmycin-resistant strain *X. oryzae* (Xoo-Rzs) and zhongshengmycin-sensitive strain *X. oryzae* (Xoo-S) to investigate the metabolic signature of ZS-resistant *X. oryzae*. Our research not only revealed the metabolomic mechanisms of antibiotics resistance in Xoo-Rzs but also provided a novel method for the control of antibiotics-resistant *X. oryzae*.

## 2. Results

### 2.1. Phenotypes of Xoo-Rzs

**Figure 1:**
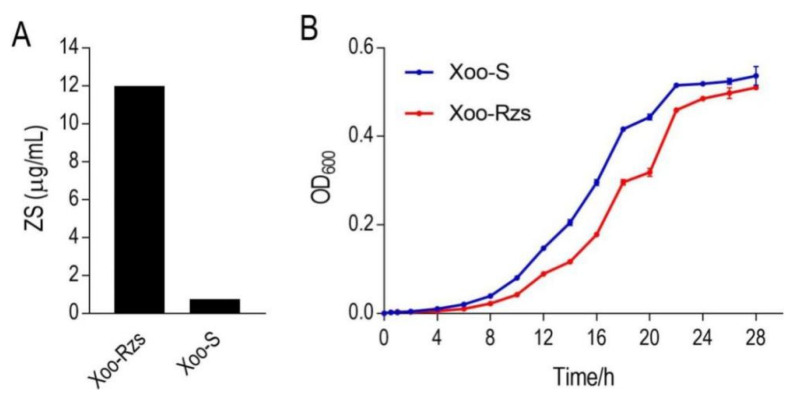
Phenotypes of Xoo-S and Xoo-Rzs. (**A**) MIC of zhongshengmycin (ZS) in Xoo-S and Xoo-Rzs. Xoo-S and Xoo-Rzs were incubated with PSA broth containing ZS ranging from 0 to 24 µg/mL in 24-well plates at 30 °C for 18 h. (**B**) Growth curve of Xoo-S and Xoo-Rzs. Xoo-S and Xoo-Rzs were diluted to 1:100 using fresh PSA medium and grown in the indicated periods. The OD_600_ of the bacterial cultures was measured at the indicated time. Note: The results in (**B**) are displayed as mean ± SD. At least three biological repeats were performed.

In order to investigate the resistance mechanism of *Xanthomonas oryzae* to ZS, we established a ZS-resistant *X. oryzae*, i.e., Xoo-Rzs, which exhibited 16-fold higher MIC levels than the parental strain, i.e., *X. oryzae* (Xoo-S) (Figure 1A). The growth rates of Xoo-Rzs were lower than those of Xoo-S during 0–16 h, but the total production showed no significant difference between the two strains after a 28 h culture (Figure 1B), and the growth rates of the two strains at specific timing points were shown in Table S2 (Supplementary Materials).

### 2.2. Difference in Metabolic Spectrum between Xoo-Rzs and Xoo-S

**Figure 2:**
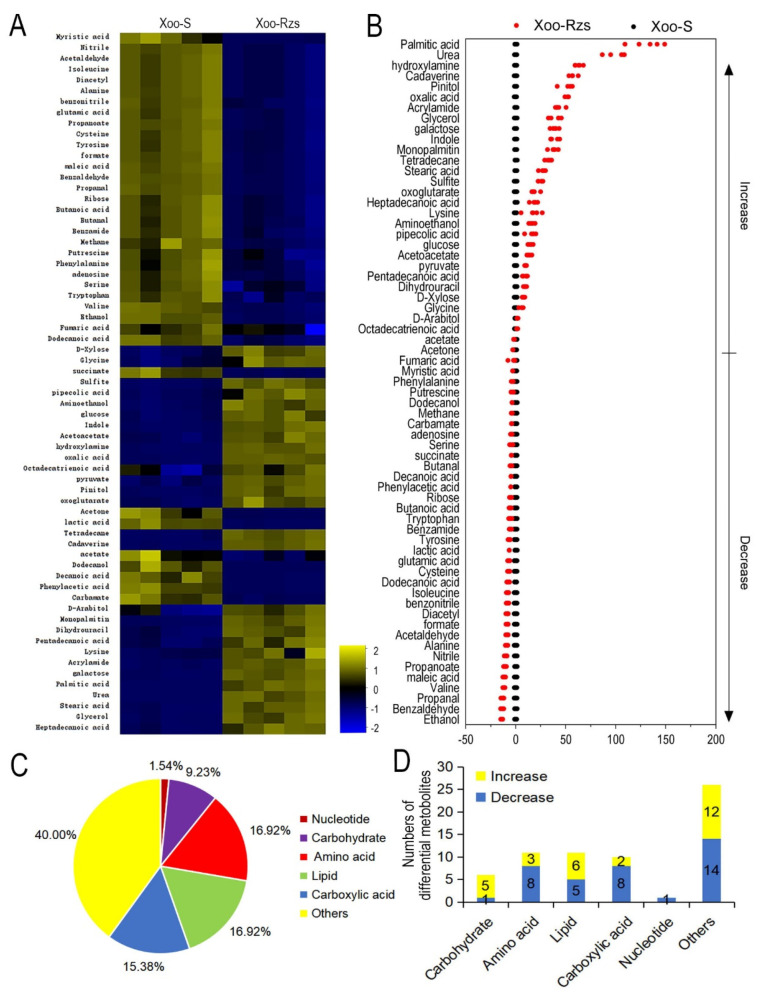
Metabolic profiles of Xoo-S and Xoo-Rzs. (**A**) Heat map of differentially abundant metabolites. Yellow and blue indicate an increase and decrease in the metabolites scaled to the mean and SD of the metabolite row, respectively (see color scale). Five biologic replicates in each group were performed, yielding a total of 10 datasets. (**B**) Z-score. The data from Xoo-Rzs are separately scaled to the mean and SD of Xoo-S. Each point represents one metabolite in one biological repeat and is colored by sample types (black: Xoo-S, red: Xoo-Rzs). (**C**) Percentage of differentially abundant metabolites in every category. (**D**) The numbers of differentially abundant metabolites increased and decreased in every category.

In order to gain insight into the metabolic alterations in Xoo-Rzs, Xoo-Rzs and Xoo-S were cultured and collected for GC–MS analysis. The bacteria were incubated into 3 mL PSA broth as a seed culture for 24 h at 30 °C, and then transferred to 50 mL PSA for enlarging cultivation. The sampling point was approximately 48 h, until the OD_600_ of the cultures reached 1.0. Five biological replicates for each strain yielded a total of 10 datasets. After removing the internal standard, i.e., ribitol, and combining the same compounds, a total of 70 metabolites were identified (Figure S1A). The categories of the identified metabolites were investigated using the Kyoto Encyclopedia of Genes and Genomes (KEGG). Six categories were enriched, including carbohydrate (10.00%), amino acid (20.00%), carboxylic acid (14.29%), lipid (17.14%), nucleotide (1.43%), and others (37.14%) (Figure S1B). The Mann–Whitney test was used to compare the metabolite abundance between Xoo-Rzs and Xoo-S. In total, 65 metabolites of differential abundance were identified and displayed as a heat map (Figure 2A). The Z values ranged from −13.65 to 131.50; the abundance of 37 metabolites was decreased and the abundance of 28 metabolites was increased in Xoo-Rzs, as compared to Xoo-S (Figure 2B). We further analyzed the differential abundances of metabolites to obtain metabolic categories. They were divided into six categories, including carbohydrate (9.23%), amino acid (16.92%), carboxylic acid (15.38%), lipid (16.92%), nucleotide (1.54%), and others (40.00%) (Figure 2C). The upregulation and downregulation of each metabolic category is shown in Figure 2D. These results indicate that the metabolism of Xoo-Rzs shifted substantially.

### 2.3. Enrichment of Metabolic Pathways in Xanthomonas Oryzae

**Figure 3:**
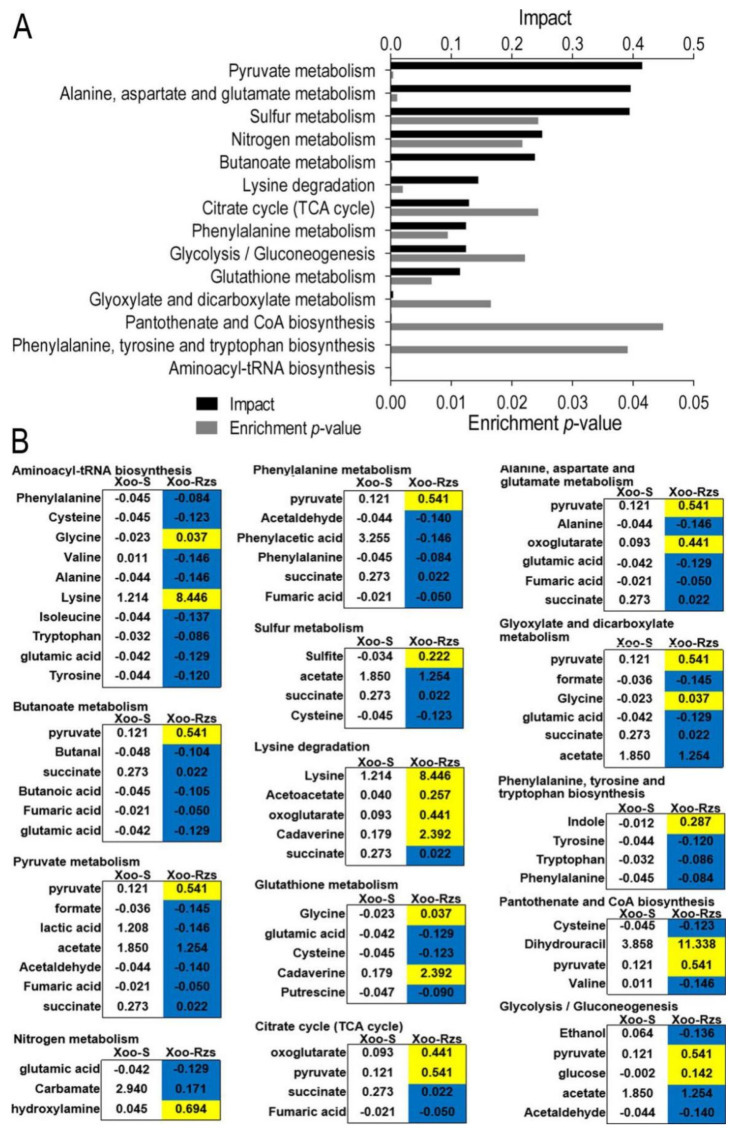
Pathway analysis. (**A**) Pathway enrichment of differentially abundant metabolites. (**B**) Integrative analysis of differentially abundant metabolites in significantly enriched pathways. Yellow and blue indicate the increase and decrease in metabolites, respectively. The numbers show the relative abundance of metabolites in Xoo-S or Xoo-Rzs.

In order to better understand the metabolic differences between Xoo-Rzs and Xoo-S, MetaboAnalyst (http://www.metaboanalyst.ca, accessed on 11 August 2021) was used to enrich the pathways of differential metabolites. Fourteen key metabolic pathways were identified: aminoacyl-tRNA biosynthesis; pyruvate metabolism; alanine, aspartate, and glutamate metabolism; butanoate metabolism; glutathione metabolism; glyoxylate and dicarboxylate metabolism; nitrogen metabolism; glycolysis/gluconeogenesis; sulfur metabolism; pantothenate and CoA biosynthesis; phenylalanine metabolism; lysine degradation; phenylalanine, tyrosine, and tryptophon biosynthesis; and the citrate cycle (TCA cycle) (Figure 3A). The relative abundances of the metabolites in each pathway are shown in Figure 3B.

### 2.4. Biomarkers of ZS Resistance

**Figure 4:**
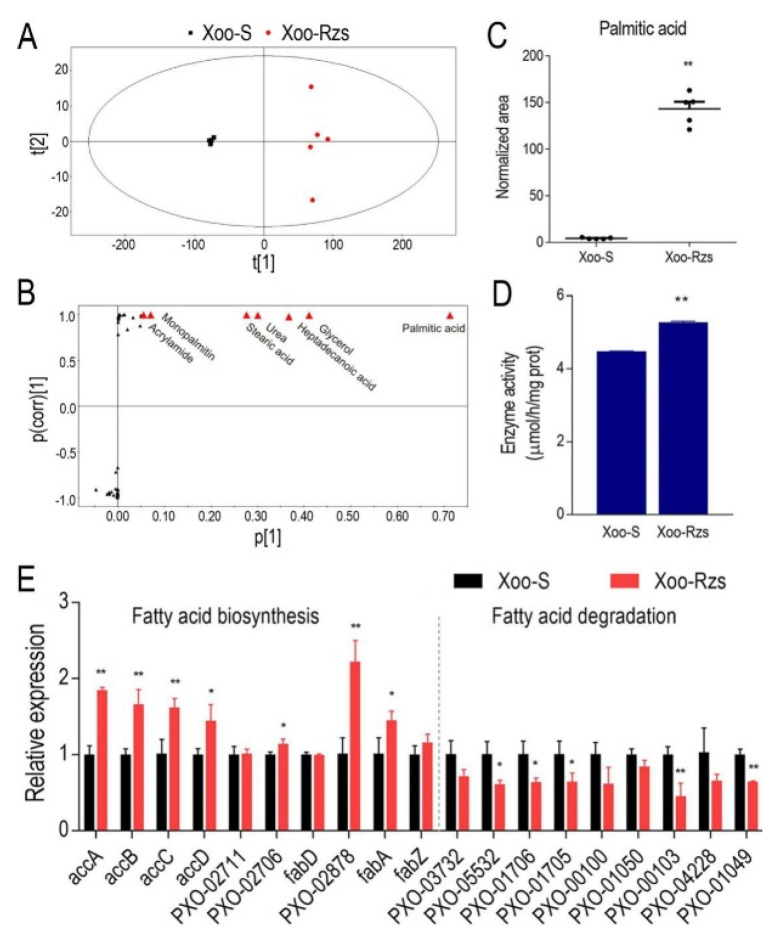
Biomarkers and the altered fatty acid biosynthesis and degradation in Xoo-Rzs. (**A**) Principal component analysis of Xoo-S and Xoo-Rzs. Each dot represents one biological repeat in the plot. (**B**) S-plot generated from the orthogonal partial least-square discriminant analysis. Predictive component *p* [1] and correlation *p* (corr) [1] differentiate Xoo-S and Xoo-Rzs. Each triangle represents one differential metabolite, and the biomarkers are highlighted in red. (**C**) The relative abundance of the palmitic acid in Xoo-S and Xoo-Rzs. (**D**) Activity of Acetyl CoA carboxylase (ACC) in Xoo-S or Xoo-Rzs. (**E**) qRT-PCR for the expression of genes in fatty acid biosynthesis and degradation in Xoo-S or Xoo-Rzs. accA/B/C/D: genes encoding ACC; PXO-02711 and PXO-02706: genes encoding *β*-ketoacyl-ACP synthetase; fabD: gene encoding ACP-malonyl-transferase; PXO-02878: gene coding for *β*-ketoacyl-ACP reductase; fabA/Z: genes encoding *β*-hydroxyacyl-ACP dehydratase; PXO-03732: gene encoding fatty acyl-CoA synthetase; PXO-05532, PXO-01706, PXO-01705 and PXO-00100: genes encoding enoyl-CoA hydratase; PXO-01050 and PXO-00103: genes encoding hydroxyacyl-CoA dehydrogenase; PXO-04228 and PXO-01049: genes encoding *β*-ketoacyl-CoA thiolase. Note: Results are displayed as mean ± SD, and significant differences are identified as determined by Student’s *t*-test (* *p* < 0.05, ** *p* < 0.01). At least three biological repeats were carried out.

In order to identify the biomarkers of ZS resistance, an orthogonal partial least-square discriminant analysis (OPLS-DA) was used to identify the metabolomic profiles of Xoo-Rzs and Xoo-S. The component (t [1]) distinguished between Xoo-Rzs and Xoo-S (Figure 4A). An S-plot analysis was used to distinguish between the variables. The absolute values of the covariance *p* and correlation *p* (corr) were set to ≥0.05 or ≥0.5, respectively. According to the S-plot analysis, seven metabolites were selected as biomarkers, namely: acrylamide, monopalmitin, stearic acid, urea, heptadecanoic acid, glycerol, and palmitic acid (Figure 4B, Figure S2). Among these biomarkers, stearic acid and palmitic acid are involved in fatty acid biosynthesis. More importantly, the abundance of palmitic acid was mostly significantly upregulated in Xoo-Rzs (Figure 4C). To further demonstrate the elevated fatty acid biosynthesis, we determined the activity of acetyl CoA carboxylase (ACC), a key enzyme involved in fatty acid biosynthesis. The ACC activity in Xoo-Rzs was significantly increased as compared to Xoo-S (Figure 4D). We further used qRT-PCR to quantify the expression of 10 genes related to fatty acid biosynthesis and nine genes related to fatty acid degradation. Among them, seven genes involved in fatty acid biosynthesis were significantly upregulated, whereas five genes involved in fatty acid degradation were significantly downregulated, indicating that fatty acid biosynthesis was upregulated in Xoo-Rzs (Figure 4E). Therefore, fatty acid biosynthesis may be enhanced to resist ZS.

### 2.5. Increased Fatty Acid Biosynthesis in Xoo-Rzs

**Figure 5:**
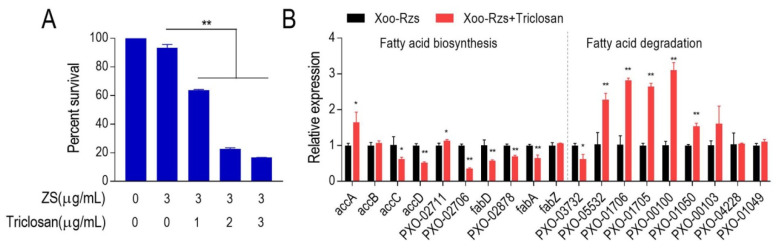
Fatty acid biosynthesis metabolism is upregulated in Xoo-Rzs. (**A**) Percent survival of Xoo-Rzs in the presence or absence of triclosan. Xoo-Rzs were incubated with ZS (3 μg/mL) plus triclosan (1, 2, and 3 μg/mL) in M9 minimal medium plus NaAc (10 mM), MgSO_4_ (2 mM), and CaCl_2_ (0.1 mM) at 30 °C for 15 h. (**B**) Expression of fatty acid biosynthesis-relatedgenes in Xoo-Rzs in the presence or absence of 1.5 µg/mL triclosan. accA/B/C/D: genes encoding ACC; PXO-02711 and PXO-02706: genes encoding *β*-ketoacyl-ACP synthetase; fabD: gene encoding ACP-malonyl-transferase; PXO-02878: gene coding for *β*-ketoacyl-ACP reductase; fabA/Z: genes encoding *β*-hydroxyacyl-ACP dehydratase; PXO-03732: gene encoding fatty acyl-CoA synthetase; PXO-05532, PXO-01706, PXO-01705 and PXO-00100: genes encoding enoyl-CoA hydratase; PXO-01050 and PXO-00103: genes encoding hydroxyacyl-CoA dehydrogenase; PXO-04228 and PXO-01049: genes encoding *β*-ketoacyl-CoA thiolase. Note: Results are displayed as mean ± SD, and significant differences are identified as determined by Student’s *t*-test (* *p* < 0.05, ** *p* < 0.01). At least three biological repeats were performed.

To explore the influence of elevated fatty acid biosynthesis on ZS resistance, we inhibited biosynthesis with the aim of detecting the ZS-mediated killing efficacy towards Xoo-Rzs. When triclosan was used, which is an inhibitor of fatty acid biosynthesis, the ZS-mediated killing was increased in a triclosan dose-dependent manner (Figure 5A). Consistently, the qRT-PCR analysis showed that the expression of genes encoding fatty acid biosynthesis was inhibited. Specifically, among the 10 genes detected, 6 were decreased, 2 were increased, and 2 remained unchanged in the presence of triclosan (Figure 5B). However, among the nine genes related to fatty acid degradation, five were increased, one was decreased, and three remained unchanged in the presence of triclosan (Figure 5B). These results indicate that the upregulation of fatty acid biosynthesis and the downregulation of fatty acid degradation is related to resistance to ZS in *X. oryzae*, and the inhibition of biosynthesis enhances ZS-mediated killing.

### 2.6. Exogenous Palmitic Acid Promotes the Resistance of Xanthomonas Oryzae to ZS

**Figure 6:**
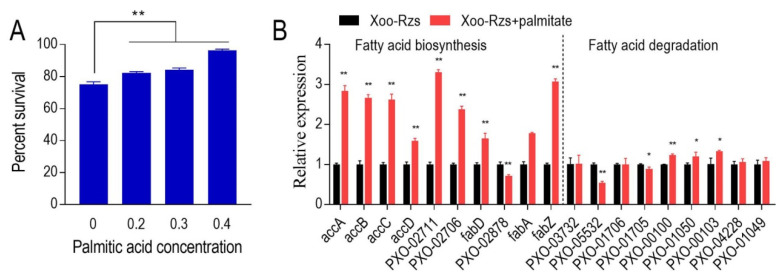
Exogenous palmitic acid promotes ZS resistance in Xoo-Rzs. (**A**) Percent survival of Xoo-Rzs in the presence or absence of palmitic acid. Xoo-Rzs were inoculated in M9 minimal medium with ZS (6 μg/mL) plus palmitic acid (0 Mm, 0.2 mM, 0.3 mM, or 0.4 mM) and incubated at 30 °C for 6 h. (**B**) Expression of fatty acid-related genes in Xoo-Rzs in the presence or absence of 0.4 mM palmitic acid. accA/B/C/D: genes encoding ACC; PXO-02711 and PXO-02706: genes encoding *β*-ketoacyl-ACP synthetase; fabD: gene encoding ACP-malonyl-transferase; PXO-02878: gene coding for *β*-ketoacyl-ACP reductase; fabA/Z: genes encoding *β*-hydroxyacyl-ACP dehydratase; PXO-03732: gene encoding fatty acyl-CoA synthetase; PXO-05532, PXO-01706, PXO-01705 and PXO-00100: genes encoding enoyl-CoA hydratase; PXO-01050 and PXO-00103: genes encoding hydroxyacyl-CoA dehydrogenase; PXO-04228 and PXO-01049: genes encoding *β*-ketoacyl-CoA thiolase. Note: Results are displayed as mean ± SD, and significant differences are identified as determined by Student’s *t*-test (* *p* < 0.05, ** *p* < 0.01). At least three biological repeats were performed.

Logically, fatty acid biosynthesis promotion can increase bacterial resistance to ZS. Therefore, exogenous palmitic acid was used to test whether the addition increases the viability of Xoo-Rzs in the presence of ZS. Indeed, the viability of the *X. oryzae* was higher in the medium with palmitic acid than without the exogenous fatty acid (Figure 6A). Consistently, palmitic acid promoted the expression of genes encoding fatty acid biosynthesis and fatty acid degradation. However, the elevated genes encoding fatty acid biosynthesis had a higher expression than the increased genes encoding fatty acid degradation (Figure 6B). These results further support the conclusion that elevated fatty acid biosynthesis enhances ZS resistance.

## 3. Discussion

The widespread use of antibiotics poses new challenges to agriculture, the environment, and human health. Therefore, it is of vital importance to study antibiotic resistance in bacteria, including agricultural crop pathogens, to ensure food safety and human and ecological health. Recent studies have shown that bacterial resistance is influenced by bacterial metabolism, and the metabolome of antibiotic-resistant bacteria can be reprogrammed by certain key biomarkers to restore bacterial sensitivity to antibiotics [38,39]. Therefore, it is important to study the metabolic mechanism of bacterial resistance and identify potential biomarkers in bacteria. The aim of this study was to explore the metabolic mechanism of Xoo-Rzs. To the best of our knowledge, this is the first study to explore the resistance mechanism of *Xanthomonas oryzae* from the perspective of metabolism.

The present study demonstrates that Xoo-Rzs has a differential metabolome in which the most prominent characteristic is the upregulation of fatty acid biosynthesis. The elevation was evidenced by the enhanced ACC activity and the transcriptional level of the genes encoding the pathways. This finding is consistent with recent studies that report that antibiotic-resistant bacteria have an antibiotic-resistant metabolome [40,41]. Therefore, metabolomics approaches can display global metabolic modulation and provide interesting avenues for further investigation.

To explore whether enhanced fatty acid biosynthesis is related to ZS resistance, two methods were adopted. Firstly, fatty acid biosynthesis was inhibited by triclosan to investigate whether the ZS-mediated killing of Xoo-Rzs was elevated. Secondly, fatty acid biosynthesis was promoted by palmitic acid to investigate whether the ZS-mediated killing of Xoo-Rzs was decreased. Our results showed that triclosan potentiated ZS to kill Xoo-Rzs, while palmitic acid inhibited the ZS-mediated killing. The potentiation and inhibition were accompanied by the elevation and decreased expression of genes encoding fatty acid biosynthesis, respectively. The association of elevated fatty acid synthesis and antibiotic resistance has been previously reported [26,27,42]; however, the relationship between increased fatty acid biosynthesis and *X. oryzae* resistance to ZS is a novel finding.

The effect of key metabolites on bacterial sensitivity and resistance to antibiotics has been reported [43,44,45]. Studies demonstrated that exogenous additives regulate the resistance of drug-resistant bacteria. Six exogenous metabolites reprogram the colicin-resistant metabolome into a colicin-sensitive metabolome, resulting in increased gene expression, enzyme activity, and/or protein abundance, thereby elevating the sensitivity to the drug [46]. Glucose, fructose, and/or alanine transform kanamycin-resistant bacteria into kanamycin-sensitive bacteria to potentiate kanamycin-mediated killing [47]. Exogenous glucose restores the sensitivity of *Vibrio alginolyticus* to gentamicin [48]. Metabolites in the TCA cycle promote *Edwardsiella tarda* resistance to chloramphenicol [49]. In this study, exogenous palmitic acid promoted the resistance of *X. oryzae* to ZS, whereas triclosan potentiated ZS to kill Xoo-Rzs. These findings show that elevated fatty acid biosynthesis is required for ZS resistance. Therefore, comprehensive metabolic analyses will be helpful in understanding the mechanisms of antibiotic resistance and identifying an approach with which to control antibiotic-resistant bacteria via metabolic modulation.

## 4. Materials and Methods

### 4.1. Strain Characteristics and MIC Measurement

The wild-type strain of *Xanthomonas oryzae* used in this study was a Zhongshengmycin (ZS)-sensitive strain (Xoo-S herein), and Xoo-Rzs refers to the *X. oryzae* strain selected by antibiotic-stressed subculturing, which harbors high resistance to ZS. PSA medium (peptone 10 g/L, sucrose 10 g/L, sodium glutamate 1 g/L, agar 2%, pH = 7.0) was used for initial culture, and PSA broth (PSA medium without agar) was used for rapid growth to create the growth curves for the strains.

For the minimum inhibitory concentration (MIC) measurement, 1% (*v*/*v*) Xoo-S and Xoo-Rzs were inoculated into 2 mL PSA broth with different concentrations of ZS (concentration gradient of 0, 0.375, 0.75, 1.5, 3, 6, 12, and 24 µg/mL) in 24-deep-well plates and cultured at 600 rpm, 30 °C. The OD_600_ value was measured after 18 h of shaking using the Multiskan Sky microplate reader, and the minimum concentration of ZS that inhibited strain growth was defined as the MIC.

### 4.2. Sample Preparation and Parameter Setting for the GC–MS Analysis

*X. oryzae* (1%) was inoculated into 50 mL PSA broth and cultured at 30 °C, 200 rpm to the value of OD_600_ = 1.0. An appropriate amount of *X. oryzae* culture was collected and centrifuged at 4 °C and 10,000 rpm for 5 min. The supernatant was discarded, and the bacterial cells were washed with 0.9% saline three times. Thereafter, 10 mL of saline was added, and a 2-fold volume of frozen methanol (placed at −20 °C for more than 24 h) was immediately added to quench the sample for 1 h at 4 °C to terminate cell metabolism. Bacterial cells were collected after centrifugation at 12,000 rpm for 5 min at 4 °C, followed by resuspension with 500 μL frozen chromatographic grade methanol. A total of 10 μL ribitol with a concentration of 0.2 mg/mL was used as an internal reference. The cells were lysed with sound waves (60% intensity) for 6 min and centrifuged at 4 °C, 12,000× *g* for 10 min. A total of 500 μL supernatant was transferred to a new centrifuge tube and dried with a nitrogen blower. Thereafter, 80 μL of methoxyamine hydrochloride (20 mg/mL) was added to the sample, followed by shaking at 200 rpm, 37 °C for 30 min to expose the protected carbonyl part of the sample. Then, 80 μL of *N*-methyl-n-trimethylsilyl trifluoroacetamide (MSTFA, Sigma-Aldrich, Shanghai, China) was added and reacted at 37 °C for 30 min for the acidic protons-derived. Finally, the samples were centrifuged at 4 °C for 5 min at 12,000× *g* and prepared to be processed on the GC–MS. The GC–MS analysis was performed following a two-stage technique as previously described [50,51]. Briefly, the injection port was maintained at 270 °C. Then, 1 μL derivatized sample was injected into a Rxi-5MS column (30 m × 250 μm i.d. × 0.25 μm) using splitless injection, and the analysis was carried out using an GC-GC HRT 4D plus (LECO, St. Joseph, MI, USA). The initial temperature of the GC oven was held at 85 °C for 3 min, followed by an increase to 285 °C at a rate of 5 °C/min, then a rise to 310 °C at a rate of 20 °C/min where it was held for 7 min. Helium was used as the carrier gas, and flow was kept constant at 1 mL/min. The MS was operated by using EI source, 70 eV EM voltage, and the scanning range was from 35 to 600 amu.

### 4.3. Technical Tools and Software Used for Data Management

The relative abundance of a metabolite was scaled according to the total abundance of all metabolites in the sample. The Z-score analysis scaled each metabolite according to a reference distribution and was calculated based on the mean and SD of the reference. Hierarchical clustering was completed in the R platform with the gplots package using the distance matrix. Principal component analysis (SIMCA-P 12.0) was used to discriminate sample patterns to identify the metabolites in Xoo-Rzs and to minimize the interindividual variation’s influence. SPSS Statistics and Graphpad Prism 7 Project were used to draw the histogram, the scatter plot, and other graphs. In this study, the method of data analysis was multivariate statistical analysis [39,47]. The specific method was performed as previously described [52].

### 4.4. Determination of the Activity of ACC

*X. oryzae* (1%) was inoculated into 30 mL PSA broth and cultured at 30 °C, 200 rpm for 24 h. An appropriate amount of *X. oryzae* culture was collected and centrifuged at 4 °C and 10,000 rpm for 5 min. The supernatant was discarded and an appropriate amount of extract (in the ratio of 5 million cells to 1 mL of extract) was added. The cells were lysed with sound waves (60% intensity) for 6 min and centrifuged at 4 °C, 12,000× *g* for 10 min, and the supernatant was used as the sample. The acetyl CoA carboxylase (ACC) activity was measured with the Acetyl CoA carboxylase Assay Kit (Suzhou geruisi biotechnology company, Suzhou, China). The protein concentration was determined using a BCA Protein Assay Kit (Dalian meilunbio biotechnology company, Dalian, China).

### 4.5. Assay of the Gene Expression Levels In Vivo

The expression levels of the genes related to fatty acid biosynthesis and degradation metabolism were detected using quantitative real-time PCR (qRT-PCR) following a method described previously [53]. In brief, the bacterial cells were harvested and the total RNA of each sample was isolated with a Biospin Total RNA Extraction Kit (Dobiothec, Fuzhou, China). An amount of 1 mg of total RNA was used for reverse transcription with the TransScript One-Step gDNA Removal and cDNA Synthesis Supermix Kit (TransGen Biotech, Beijing, China). qRT-PCR was performed in 96-well plates with PerfectStart Green qPCR SuperMix (TransGen Biotech, Beijing, China). The primers for each gene were listed in Table S1. Each primer pair was specific, and the relative expression of each gene was determined by the comparative threshold cycle method (2-ΔΔCT method).

### 4.6. Antibiotic Bactericidal Assay

Xoo-Rzs was cultured in PSA broth for 24 h at 30 °C. Samples were harvested at 8000 rpm for 3 min centrifugation, and the cells were washed three times with 30 mL sterile saline. The cells were transferred into M9 broth (NaAc 10 mM, MgSO_4_ 2 mM, and CaCl_2_ 0.1 mM) containing the indicated concentration of ZS. To test the effect of triclosan on the antibiotic resistance of Xoo-Rzs, 3 μg/mL ZS and 1–3 μg/mL triclosan were added in the M9, while, to test the effect of palmitic acid, the concentration of ZS was 6 μg/mL, and the concentrations of palmitic acid were 0.2, 0.3, or 0.4 mM. The OD_600_ of the suspension was adjusted to 0.2, and the exogenous reagents, i.e., triclosan or palmitic acid, were added to a final volume of 5 mL. After incubation at 30 °C for 6 h, 100 µL aliquot samples were collected, serially diluted, and plated (10 µL aliquots) on PSA medium, then cultured at 30 °C for 48–60 h for the CFU calculation. Only those dilutions generating 20–200 clones per plate were counted. Percent survival was determined by dividing the CFU obtained from a treated sample by the CFU obtained from the control.

## 5. Conclusions

In conclusion, *X. oryzae* resistance to ZS is related to an increase in fatty acid biosynthesis. The use of triclosan to inhibit fatty acid metabolism enhances the sensitivity of *X. oryzae* to ZS, while exogenous palmitic acid weakens its sensitivity to ZS. Our study showed the importance of fatty acid biosynthesis in Xoo-Rzs resistance to ZS. These results will deepen our understanding of the mechanisms through which antibiotic resistance forms in bacteria from a metabolic perspective. Furthermore, they provide new strategies for rational drug use, which is important for preventing and reducing bacterial antibiotic resistance.

## Data Availability

Data is contained within the article.

## Supplementary Materials

The following are available online at https://www.mdpi.com/article/10.3390/antibiotics10101166/s1, Figure S1: Total metabolites detected in Xoo-S and Xoo-Rzs, Figure S2: The relative abundances of the 6 biomarkers. Table S1: Primers for qRT-PCR. Table S2: The specific growth rate of two strains.

## References

[1] Ke Y.G., Wu M.X., Zhang Q.L., Li X.H., Xiao J.H., Wang S.P. (2019). Hd3a and OsFD1 negatively regulate rice resistance to *Xanthomonas oryzae* pv. *oryzae* and *Xanthomonas oryzae* pv. *oryzicola*. Biochem. Bioph. Res. Commun..

[2] Nino-Liu D.O., Ronald P.C., Bogdanove A.J. (2006). *Xanthomonas oryzae* pathovars: Model pathogens of a model crop. Mol. Plant Pathol..

[3] Chen X.L., Yan Q., Li R.F., Li K.H., Gao L.J. (2016). First report of a new and highly virulent race of *Xanthomonas oryzae* pv. *oryzae*, the causal agent of bacterial leaf blight of rice in Guangxi Province, China. Plant Dis..

[4] Swings J., Vandenmooter M., Vauterin L., Hoste B., Gillis M., Mew T.W., Kersters K. (1990). Reclassification of the causal agents of bacterial-blight (*Xanthomonas campestris* pv. *oryzae*) and bacterial leaf streak (*Xanthomonas campestris* pv. *oryzicola*) of rice as pathovars of *Xanthomonas Oryzae* (Ex Ishiyama 1922) sp nov, nom. rev. Int. J. Syst. Bacteriol..

[5] Gnanamanickam S.S., Priyadarisini V.B., Narayanan N.N., Vasudevan P., Kavitha S. (1999). An overview of bacterial blight disease of rice and strategies for its management. Curr. Sci. India.

[6] Yang P., Li F.J., Huang S.W., Luo M., Lin W., Yuan G.Q., Li Q.Q. (2020). Physiological and transcriptional response of *Xanthomonas oryzae* pv. *oryzae* to berberine, an emerging chemical control. Phytopathology.

[7] Mew T.W., Alvarez A.M., Leach J.E., Swings J. (1993). Focus on Bacterial-Blight of Rice. Plant Dis..

[8] Jin P.F., Wang Y., Tan Z., Liu W.B., Miao W.G. (2020). Antibacterial activity and rice-induced resistance, mediated by C(15)surfactin A, in controlling rice disease caused by *Xanthomonas oryzae* pv. *oryzae*. Pestic Biochem. Phys..

[9] Yasmin S., Hafeez F.Y., Mirza M.S., Rasul M., Arshad H.M.I., Zubair M., Iqbal M. (2017). Biocontrol of bacterial leaf blight of rice and profiling of secondary metabolites produced by rhizospheric *Pseudomonas aeruginosa* BRp3. Front Microbiol..

[10] Ham J.H., Melanson R.A., Rush M.C. (2011). Burkholderia glumae: Next major pathogen of rice?. Mol. Plant Pathol..

[11] Zhu X.F., Xu Y., Peng D., Zhang Y., Huang T.T., Wang J.X., Zhou M.G. (2013). Detection and characterization of bismerthiazol-resistance of *Xanthomonas oryzae* pv. *oryzae*. Crop Prot..

[12] Xu Y., Zhu X.F., Zhou M.G., Kuang J., Zhang Y., Shang Y., Wang J.X. (2010). Status of streptomycin resistance development in *Xanthomonas oryzae* pv. *oryzae* and *Xanthomonas oryzae* pv. *oryzicola* in China and their resistance characters. J. Phytopathol..

[13] Yi C.F., Chen J.X., Hu D.Y., Song B.A. (2020). First report about the screening, characterization, and fosmid library construction of *Xanthomonas oryzae* pv. *oryzae* strain with resistance to fubianezuofeng. Pestic Biochem. Phys..

[14] Wang Q.P., Zhang C., Long Y.H., Wu X.M., Su Y., Lei Y., Ai Q. (2021). Bioactivity and control efficacy of the novel antibiotic tetramycin against *Various Kiwifruit* diseases. Antibiotics.

[15] Ren Y., Li D., Jiang S., Wang Y., Tang Q., Huang H., Wang D., Song B., Chen Z. (2021). Integration of transcriptomic and proteomic data reveals the possible action mechanism of the antimicrobial zhongshengmycin against *Didymella segeticola*, the causal agent of tea leaf spot. Phytopathology.

[16] Wang B.L., Shi Y.X., Ma Y., Liu X.H., Li Y.H., Song H.B., Li B.J., Li Z.-M. (2010). Synthesis and biological activity of some novel trifluoromethyl-substituted 1,2,4-triazole and bis(1,2,4-Triazole) mannich bases containing piperazine rings. J. Agr. Food Chem..

[17] Wang B.L., Shi Y.X., Zhang S.J., Ma Y., Wang H.X., Zhang L.Y., Wei W., Liu X.-H., Li Y.-H., Li Z.-M. (2016). Syntheses, biological activities and SAR studies of novel carboxamide compounds containing piperazine and arylsulfonyl moieties. Eur. J. Med. Chem..

[18] Song Y., Shi J.T., Xiong Z.Z., Shentu X.P., Yu X.P. (2021). Three antimicrobials alter gut microbial communities and causing different mortality of brown planthopper, *Nilaparvata lugens* Stal. Pestic Biochem. Phys..

[19] Alekshun M.N., Levy S.B. (2007). Molecular mechanisms of antibacterial multidrug resistance. Cell.

[20] Shaw W.V. (1983). Chloramphenicol acetyltransferase: Enzymology and molecular biology. CRC Crit. Rev. Biochem..

[21] Murray I.A., Shaw W.V. (1997). O-Acetyltransferases for chloramphenicol and other natural products. Antimicrob. Agents Chemother..

[22] Gabryszewski S.J., Wong Fok Lung T., Annavajhala M.K., Tomlinson K.L., Riquelme S.A., Khan I.N., Noguera L.P., Wickersham M., Zhao A., Mulenos A.M. (2019). Metabolic adaptation in methicillin-resistant *Staphylococcus aureus* pneumonia. Am. J. Respir. Cell Mol. Biol..

[23] Wen X., Cao J., Mi J., Huang J., Liang J., Wang Y., Ma B., Zou Y., Liao X., Liang J.B. (2021). Metabonomics reveals an alleviation of fitness cost in resistant *E. coli* competing against susceptible *E. coli* at sub-MIC doxycycline. J. Hazard Mater..

[24] Liu Y., Yang K., Jia Y., Shi J., Tong Z., Wang Z. (2020). Cysteine potentiates bactericidal antibiotics activity against Gram-negative bacterial persisters. Infect. Drug Resist..

[25] Ma Y., Guo C., Li H., Peng X.X. (2013). Low abundance of respiratory nitrate reductase is essential for *Escherichia coli* in resistance to aminoglycoside and cephalosporin. J. Proteom..

[26] Liu S.R., Pen X.X., Li H. (2019). Metabolic mechanism of ceftazidime resistance in *Vibrio alginolyticus*. Infect. Drug Resist..

[27] Cheng Z.X., Yang M.J., Peng B., Peng X.X., Lin X.M., Li H. (2018). The depressed central carbon and energy metabolisms is associated to the acquisition of levofloxacin resistance in *Vibrio alginolyticus*. J. Proteom..

[28] Lee H.H., Molla M.N., Cantor C.R., Collins J.J. (2010). Bacterial charity work leads to population-wide resistance. Nature.

[29] Mishra S., Yang X.D., Ray S., Fraceto L.F., Singh H.B. (2020). Antibacterial and biofilm inhibition activity of biofabricated silver nanoparticles against *Xanthomonas oryzae* pv. *oryzae* causing blight disease of rice instigates disease suppression. World J. Microb. Biot..

[30] Majumdar T.D., Singh M., Thapa M., Dutta M., Mukherjee A., Ghosh C.K. (2019). Size-dependent antibacterial activity of copper nanoparticles against *Xanthomonas oryzae* pv. *oryzae*—A synthetic and mechanistic approach. Colloid Interfac. Sci..

[31] Antar A., Lee M.A., Yoo Y., Cho M.H., Lee S.W. (2020). PXO_RS20535, Encoding a novel response regulator, is required for chemotactic motility, biofilm formation, and tolerance to oxidative stress in *Xanthomonas oryzae* pv. *oryzae*. Pathogens.

[32] Li P.P., Liu X.J., Li H., Peng X.X. (2012). Downregulation of Na(+)-NQR complex is essential for *Vibrio alginolyticus* in resistance to balofloxacin. J. Proteom..

[33] Yang J.H., Bening S.C., Collins J.J. (2017). Antibiotic efficacy—Context matters. Curr. Opin. Microbiol..

[34] Ye J.Z., Lin X.M., Cheng Z.X., Su Y.B., Li W.X., Ali F.M., Zheng J., Peng B. (2018). Identification and efficacy of glycine, serine and threonine metabolism in potentiating kanamycin-mediated killing of *Edwardsiella piscicida*. J. Proteom..

[35] Ye J.Z., Su Y.B., Lin X.M., Lai S.S., Li W.X., Ali F., Zheng J., Peng B. (2018). Alanine enhances aminoglycosides-induced ROS production as revealed by proteomic analysis. Front Microbiol..

[36] Wu C.W., Zhao X.L., Wu X.J., Wen C., Li H., Chen X.H., Peng X.X. (2015). Exogenous glycine and serine promote growth and antifungal activity of *Penicillium citrinum* W1 from the south-west Indian Ocean. FEMS Microbiol. Lett..

[37] Wu C.W., Wu X.J., Wen C., Peng B., Peng X.X., Chen X.H., Li H. (2016). Fructose promotes growth and antifungal activity of *Penicillium citrinum*. Protein Cell.

[38] Isha A., Yusof N.A., Shaari K., Osman R., Abdullah S.N.A., Wong M.Y. (2020). Metabolites identification of oil palm roots infected with *Ganoderma boninense* using GC-MS-based metabolomics. Arab J. Chem..

[39] Wang Z., Li M.Y., Peng B., Cheng Z.X., Li H., Peng X.X. (2016). GC-MS-Based metabolome and metabolite regulation in serum-resistant *Streptococcus agalactiae*. J. Proteome Res..

[40] Chen X.H., Zhang B.W., Li H., Peng X.X. (2015). Myo-inositol improves the host’s ability to eliminate balofloxacin-resistant *Escherichia coli*. Sci. Rep..

[41] Peng B., Su Y.B., Li H., Han Y., Guo C., Tian Y.M., Peng X.X. (2015). Exogenous alanine and/or glucose plus kanamycin klls antibiotic-resistant bacteria. Cell Metab..

[42] Su Y.-B., Kuang S.-F., Ye J.-Z., Tao J.-J., Li H., Peng X.-X., Peng B. (2021). Enhanced biosynthesis of fatty acids is associated with the acquisition of ciprofloxacin resistance in *Edwardsiella tarda*. mSystems.

[43] Jiang M., Kuang S.F., Lai S.S., Zhang S., Yang J., Peng B., Peng X.X., Chen Z.G., Li H. (2020). Na(+)NQR confers aminoglycoside resistance via the regulation of L-alanine netabolism. mBio.

[44] Lin Y.X., Li W.X., Sun L.N., Lin Z.P., Jiang Y.H., Ling Y.M., Lin X. (2019). Comparative metabolomics shows the metabolic profiles fluctuate in multi-drug resistant *Escherichia coli strains*. J. Proteom..

[45] Zhang S., Yang M.J., Peng B., Peng X.X., Li H. (2020). Reduced ROS-mediated antibiotic resistance and its reverting by glucose in *Vibrio alginolyticus*. Environ. Microbiol..

[46] Li L., Su Y.B., Peng B., Peng X.X., Li H. (2020). Metabolic mechanism of colistin resistance and its reverting in *Vibrio alginolyticus*. Environ. Microbiol..

[47] Allison K.R., Brynildsen M.P., Collins J.J. (2011). Metabolite-enabled eradication of bacterial persisters by aminoglycosides. Nature.

[48] Zhang S., Wang J., Jiang M., Xu D., Peng B., Peng X.X., Li H. (2019). Reduced redox-dependent mechanism and glucose-mediated reversal in gentamicin-resistant *Vibrio alginolyticus*. Environ. Microbiol..

[49] Wang C., Dong X.S., Yang Y.Y., Xu G.J., Wu M.M., Yan F.J., Zhang L.G., An L., Fu P.S., Wang X.R. (2021). Metabolites in the TCA cycle promote resistance to chloramphenicol of *Edwardsiella tarda*. J. Proteome Res..

[50] Jiang M., Yang L.F., Zheng J., Chen Z.G., Peng B. (2020). Maltose promotes crucian carp survival against *Aeromonas sobrialinfection* at high temperature. Virulence.

[51] Zhao X.L., Wu C.W., Peng X.X., Li H. (2014). Interferon-alpha 2b against microbes through promoting biosynthesis of unsaturated fatty acids. J. Proteome Res..

[52] Guan Y., Wang Q.X., Lv C., Wang D.H., Ye X.Y. (2021). Fermentation time-dependent pectinase activity is associated with metabolomics variation in *Bacillus licheniformis* DY2. Process Biochem..

[53] Jiang M., Gong Q.Y., Lai S.S., Cheng Z.X., Chen Z.G., Zheng J., Peng B. (2019). Phenylalanine enhances innate immune response to clear ceftazidime-resistant *Vibrio alginolyticus* in Danio rerio. Fish Shellfish Immunol..

