# Supplementary material for: Elevation of Fatty Acid Biosynthesis Metabolism Contributes to Zhongshengmycin Resistance in Xanthomonas oryzae
Source: Antibiotics (Basel). 2021 Sep 25;10(10):1166. doi: 10.3390/antibiotics10101166 (PMC8532796; doi:10.3390/antibiotics10101166)
Supplement: Supplementary file 1 [file antibiotics-10-01166-s001.zip › antibiotics-1362739-supplementary.pdf]

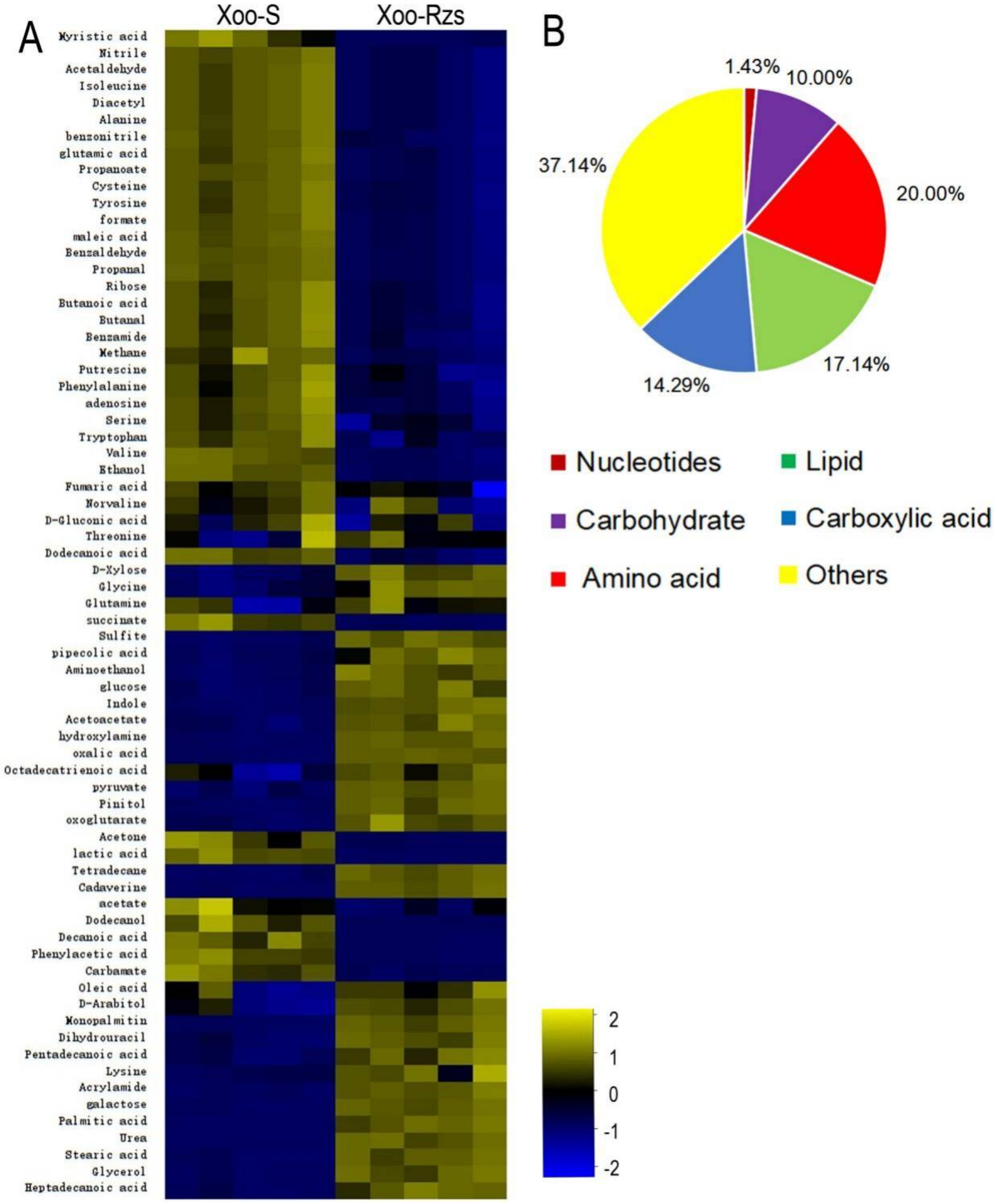

Figure S1. Total metabolites detected in Xoo-S and Xoo-Rzs. (A) Heat map of differential abundance metabolites. Yellow and blue indicate the increase and decrease of the metabolites scaled to mean and SD of row metabolite level, respectively (see color scale). (B) Percentage of the total metabolites in every category.

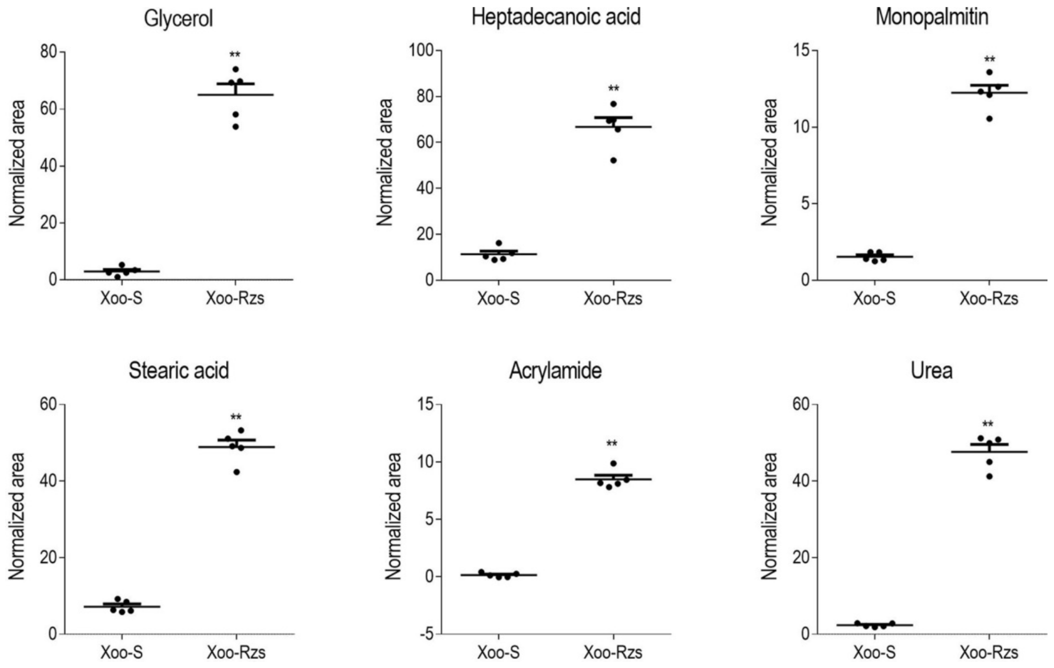

Figure S2. The relative abundances of the 6 biomarkers.

Table S1. Primers for qRT-PCR.

Genes	Primers	Sequences (5′-3′)	Annotation
qRT-16s	Forward	AACACTGACACTGAGGCACGAAAG	16s rDNA
	Reverse	CCCAGGCGGCGAACTTAACG	
accA	Forward	GAAATGGCCGAGCTGAAAATTCCG	acetyl-CoA carboxylase carboxyltransferase subunit alpha
	Reverse	AATAGGTGCCGTATTCCAGCATCAG	
accB	Forward	ATGGATCTCCGCAAAATCAAGAAACTG	acetyl-CoA carboxylase biotin carboxyl carrier protein
	Reverse	ACGCTTTCTTCGCCTTCCTTGATC	
accC	Forward	CCAGCGACAGTTACCTCAACATCC	acetyl-CoA carboxylase biotin carboxylase subunit
	Reverse	CCCGATGAAGATGAAGCCGGATTG	
accD	Forward	TGGCTCAGCAAATTGATGCCTTCC	acetyl-CoA carboxylase, carboxyltransferase subunit beta
	Reverse	CAATTGCTGCACTTTTCCCACAGG	
PXO-02711	Forward	ACCAAGATCGCCGGTGAAATCAAG	3-oxoacyl-[acyl-carrier-protein] synthase II
	Reverse	CGAGACCGTAGTGGATGAACGAATC	
PXO-02706	Forward	GCTATTTGCCCGAAAAGGTGTTGAC	3-oxoacyl-[acyl-carrier-protein] synthase III
	Reverse	GAATACCAGTGCGCGAGAAGATCC	
fabD	Forward	TGACCGAATCCACTCTCGCC	ACP S-malonyltransferase
	Reverse	GAGGCTTCGGCGAACGTTTC	
PXO-02878	Forward	TGATCGTGCATGCCAATCGC	3-oxoacyl-[acyl-carrier-protein] reductase
	Reverse	GCCAACTTCAAGCAACGCCT	
fabA	Forward	ATGACTCGTCAAAGTGCGTACTCG	beta-hydroxydecanoyl-ACP dehydratase
	Reverse	ATCAGCATCGGATCATTGGGCAAG	
fabZ	Forward	CACCGCTATCCGTTCCTGTTGATC	3-hydroxyacyl-ACP dehydratase FabZ
	Reverse	TCGTTGATGCTGACGTTCTTCTGC	
PXO-03732	Forward	TGGAGCAGCACTACAGCGTCAG	fatty acyl CoA synthetase
	Reverse	GTTCGTCCTTGCGTCCGTACAG	
PXO-05532	Forward	GCTGACACCGCCAAACCCTATG	enoyl-CoA hydratase
	Reverse	CAACAGTCGCTGGATGCCTTCC	
PXO-01706	Forward	CCGACACCTTCCGTACCGAATAC	enoyl-CoA hydratase
	Reverse	CCAGTGCGGTTGACGATCCTTG	
PXO-01705	Forward	GTGGAAGAGGTGGTTGGCAAGG	enoyl-CoA hydratase
	Reverse	ACACGCTGATTGGGCTCTGTTTAC	
PXO-00100	Forward	CGGTCATTTCGCCCTATGTGGTTG	enoyl-CoA hydratase
	Reverse	AGCATCGAAGATTTCGGCAGTGG	
PXO-01050	Forward	AAGGGTGTTGTGTATGCCAAGGAC	3-hydroxyacyl-CoA dehydrogenase
	Reverse	AATTGCTGCGTGTGGTGGATGG	
PXO-00103	Forward	GGGCTCAACCTGGTGGTGAATTG	3-hydroxyacyl-CoA dehydrogenase type II
	Reverse	ACATTGAAGCTGCCGACCAGATTC	
PXO-04228	Forward	AGCACTCCTCGGACTGGAATCTG	3-ketoacyl-CoA thiolase
	Reverse	TTGGCGATGGTAATCACCGAATCC	
PXO-01049	Forward	CGATGTCGATGGTGCCGATGATG	3-ketoacyl-CoA thiolase
	Reverse	CCTTCCATTCTTCTGCGACCTTCTC	

Table S2. The specific growth rate of two strains.

Culture Time	Specific Growth Rate	
	Xoo-S	Xoo-Rzs
0-0.5 h	0.00	0.00
0.5-1 h	0.00	0.00
1-2 h	0.00	0.00
2-4 h	0.00	0.00
4-6 h	0.01	0.00
6-8 h	0.01	0.01
8-10 h	0.02	0.01
10-12 h	0.03	0.02
12-14 h	0.03	0.01
14-16 h	0.05	0.03
16-18 h	0.06	0.06
18-20 h	0.01	0.01
20-22 h	0.04	0.07
22-24 h	0.00	0.01
26-28 h	0.00	0.01
